# Sporadic and Lynch syndrome-associated mismatch repair-deficient brain tumors

**DOI:** 10.1038/s41374-021-00694-3

**Published:** 2021-11-30

**Authors:** Hyunhee Kim, Ka Young Lim, Jin Woo Park, Jeongwan Kang, Jae Kyung Won, Kwanghoon Lee, Yumi Shim, Chul-Kee Park, Seung-Ki Kim, Seung-Hong Choi, Tae Min Kim, Hongseok Yun, Sung-Hye Park

**Affiliations:** 1 Department of Pathology, Seoul National University College of Medicine, Seoul, Republic of Korea; 2 Department of Neurosurgery, Seoul National University College of Medicine, Seoul, Republic of Korea; 3 Department of Radiology, Seoul National University College of Medicine, Seoul, Republic of Korea; 4 Department of Internal Medicine, Seoul National University College of Medicine, Seoul, Republic of Korea; 5 Department of Genomic Medicine, Seoul National University College of Medicine, Seoul, Republic of Korea; 6 Institute of Neuroscience, Seoul National University College of Medicine, Seoul, Republic of Korea

**Keywords:** Cancer genomics, Oncogenesis

## Abstract

Mismatch repair-deficient (MMRD) brain tumors are rare among primary brain tumors and can be induced by germline or sporadic mutations. Here, we report 13 MMRD-associated (9 sporadic and 4 Lynch syndrome) primary brain tumors to determine clinicopathological and molecular characteristics and biological behavior. Our 13 MMRD brain tumors included glioblastoma (GBM) *IDH*-wildtype (*n* = 9) including 1 gliosarcoma, astrocytoma *IDH*-mutant WHO grade 4 (*n* = 2), diffuse midline glioma (DMG) *H3* K27M-mutant (*n* = 1), and pleomorphic xanthoastrocytoma (PXA) (*n* = 1). Next-generation sequencing using a brain tumor-targeted gene panel, microsatellite instability (MSI) testing, Sanger sequencing for germline MMR gene mutation, immunohistochemistry of MMR proteins, and clinicopathological and survival analysis were performed. There were many accompanying mutations, suggesting a high tumor mutational burden (TMB) in 77%, but TMB was absent in one case of GBM, *IDH*-wildtype, DMG, and PXA, respectively. *MSH2*, *MLH1, MSH6*, and *PMS2* mutations were found in 31%, 31%, 31% and 7% of patients, respectively. MSI-high and MSI-low were found in 50% and 8% of these gliomas, respectively and 34% was MSI-stable. All Lynch syndrome-associated GBMs had MSI-high. In addition, 77% (10/13) had histopathologically multinucleated giant cells. The progression-free survival tended to be poorer than the patients with no MMRD gliomas, but the number and follow-up duration of our patients were insufficient to get statistical significance. In the present study, we found that the most common MMRD primary brain tumor was GBM *IDH*-wildtype. The genetic profile of MMRD GBM was different from that of conventional GBM. MMRD gliomas with TMB and MSI-H may be sensitive to immunotherapy but resistant to temozolomide. Our findings can help develop better treatment options.

## Abstract (web summary)

The authors examined 13 mismatch repair-deficient (MMRD)-associated primary brain tumors to determine molecular characteristics and biological behavior. MMRD occurred after chemotherapy and radiotherapy in two cases. The genetic profile of MMRD glioblastoma was different from that of conventional glioblastoma. Half of the MMRD-gliomas and all Lynch syndrome-associated GBMs were high in microsatellite instability. These tumors had high mutational burdens and tended to have shorter progression-free survival than glioma without MMRD.

## Introduction

The DNA mismatch repair (MMR) system is responsible for the prevention of genomic instability in cells and is controlled by MMR genes. Those are *mutL homolog 1* (*MLH1*), encoded at chromosome 3p21.3, *mutS homolog 2 (MSH2)* at chromosome 2p22–21, *mutS homolog 6* (*MSH6*) at chromosome 2p16 and *postmeiotic segregation increased 2 (PMS2)* at chromosome 7p22.2. MMR deficiency (MMRD) can be caused by germline or sporadic mutations or promoter methylation of MMR genes, which is associated with microsatellite instability (MSI) and tumor mutational burden (TMB). Therefore, it contributes to tumorigenesis, poor outcomes, and acquired drug resistance to alkylating agents that mediate the formation of O^6^ methylguanine-containing mismatches^1,2^. TMB is considered a potential biomarker for immune checkpoint therapy^3,4^.

Lynch syndrome is an autosomal dominant hereditary cancer syndrome that was originally reported by Warthin in 1913 and is also known as hereditary nonpolyposis colorectal cancer syndrome^5–7^. Lynch syndrome and constitutional MMRD syndrome are caused by heterozygous and homozygous germline mutations in one of the MMR genes, respectively^8,9^. Germline mutations in *MSH2* (40–50%) and *MLH1* (30–37%) are the most frequent, and *MSH6* and *PMS2* mutations are found in 7–13% and up to 9% of cases, respectively^9,10^. Patients with Lynch syndrome have a lifetime risk of 50–80% for developing colorectal cancer and 40–60% for developing endometrial cancer and less commonly cancers of the upper urinary tract, hepatobiliary tract, small intestine, ovary, and skin^11–14^. Lynch syndrome also quadruples the risk of brain tumors, predominantly high-grade gliomas (HGGs)^11–13^. For an accurate diagnosis of sporadic and hereditary MMRD tumors, immunohistochemistry (IHC) of MMR proteins in the tumors, molecular studies to detect MMR gene mutations or methylation, MSI, and genetic testing of affected family members are required^15,16^.

Lynch syndrome-associated MMRD tumors often exhibit a MSI-H phenotype^17^. However, since MSI-H is also frequently observed in sporadic colorectal cancers, genetic testing for germline MMR genes is essential^10^.

Although MMRD is well recognized in colorectal and endometrial carcinomas, MMRD brain tumors remain poorly understood. Here, we report patients with nine sporadic MMR mutation- and four Lynch syndrome-associated HGGs with MSI-H and high TMB.

## Materials and methods

### Case summary

Among 740 brain tumors from the archives of the Department of Pathology, Seoul National University Hospital, archived from 2018 to 2021 that were subjected to next-generation sequencing (NGS), 13 MMRD brain tumors were found. The tumors included glioblastoma (GBM) *IDH*-wildtype (*n* = 9), including one gliosarcoma, astrocytoma *IDH*-mutant WHO grade 4 (*n* = 2), diffuse midline glioma (DMG) *H3* K27M-mutant (*n* = 1) and pleomorphic xanthoastrocytoma (PXA) (*n* = 1). The proportion of MMRD primary brain tumors in our hospital, including cases of MMRD-associated pineal teratocarcinoma (*n* = 1) and meningioma (*n* = 1) that were not included in this study, was ~2.0%. The age of the 13 patients ranged from 11 to 78 years (median age: 50 years), and the male-to-female ratio was 1.2:1.

Ten patients had recurrent gliomas. As a result of the NGS study of the initial tumors, two of them were found to have developed MMRD after concurrent chemotherapy and radiotherapy (CCRT) (Supplementary Tables 1 and 4).

Four patients had Lynch syndrome, confirmed by the germline Sanger sequencing, but the concurrent malignancy was found in two patients who had histories of extracrainal cancers. The #4 patient had Lynch syndrome with multiple cancers; he was diagnosed with prostatic adenocarcinoma (Gleason score 8) at the age of 61 years and colonic and jejunal cancers at the age of 62 years. His colonic tumors showed a mucinous subtype in the mid-ascending colon and poorly differentiated adenocarcinoma with signet ring cell features in the proximal ascending colon and the jejunum. These subtypes and locations of intestinal adenocarcinoma are known to be associated with Lynch syndrome^18^. Immunohistochemically, the ascending colonic and jejunal adenocarcinomas showed a loss of MSH2 and MSH6 proteins in the tumor cell nuclei, but the MLH1 and PMS2 proteins were retained. Interestingly, prostatic adenocarcinoma is not an MMRD tumor with retained expression of all four MMR proteins.

**Fig. 1:**
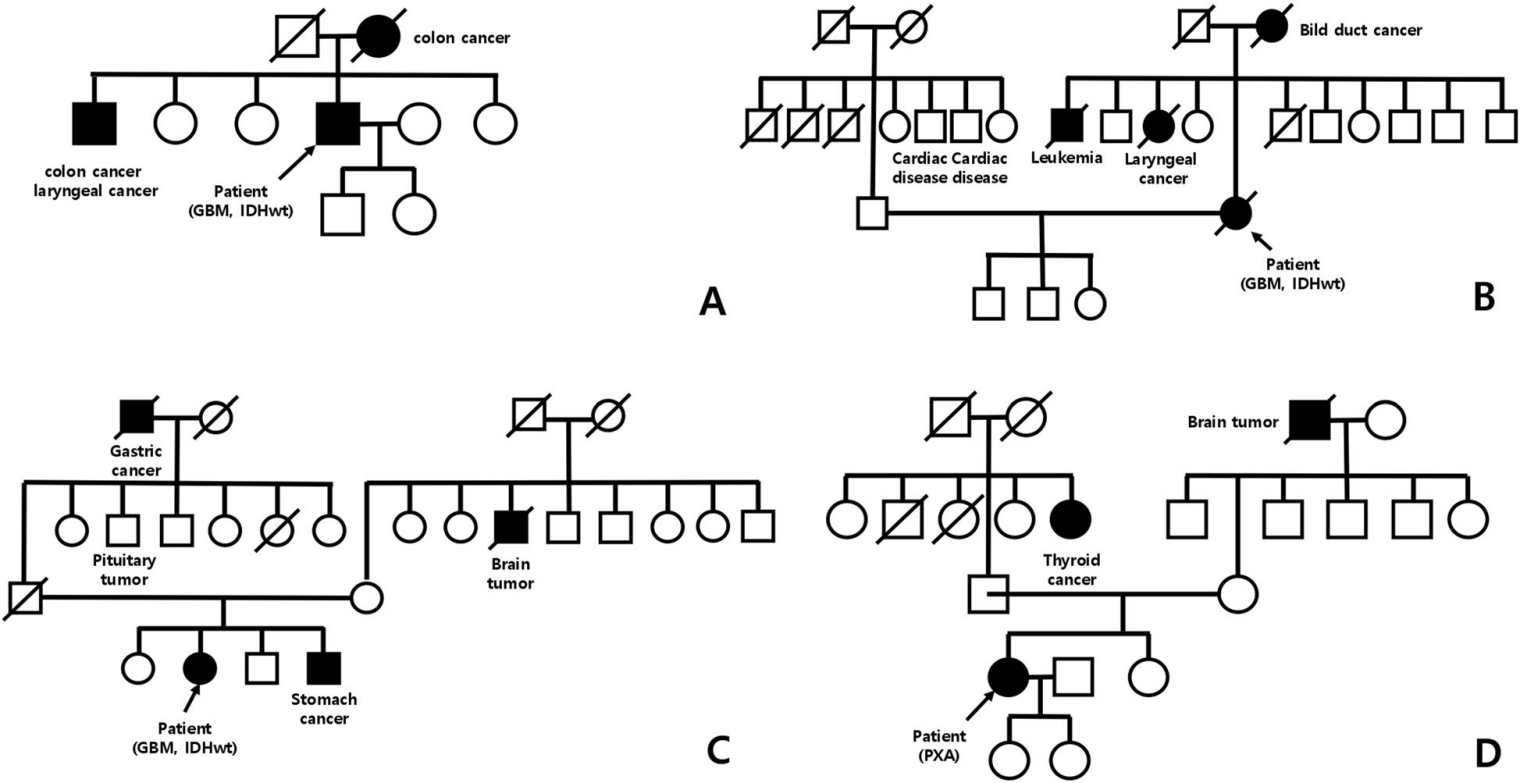
The pedigrees of four patients with Lynch syndrome. **A** The pedigree of Case 4 showing affected family members with colon cancer or laryngeal cancer. **B** The pedigree of Case 3 showing affected family members with bile duct cancer or laryngeal cancer or leukemia. **C** The pedigree of Case 8 showing affected family members with gastric cancer or brain tumor. **D** The pedigree of Case 13 showing affected family members with thyroid cancer or brain tumor.

The pedigree chart of these four patients with Lynch syndrome suggested an autosomal dominant inheritance of the disease (Fig. 1). All patients underwent craniotomy and tumor resection. Clinical manifestations are summarized in Table 1.

**Table 1:** Summary of the clinicopathologic feature of presenting patients with MMR deficient brain tumors.

#	Age	Sex	Diagnosis	Accompanying tumor	Site	Tumor size (cm)	MRI finding	Op	Postoperative treatment	Follow up	Presence of giant cells	MVP/necrosis	Ki67 index
1	47	F	GBM IDH-wt, rec	–	Lt. temporal	5	Rim enhancing mass and hemorrhage	GTR	CCRT/TMZ	Recur and died in 57 months after GTR	No (lipidized cells)	+/−	15.7%
2	41	F	GBM IDH-wt, rec	–	Rt. parietal	5.7	Heterogeneously enhancing mass	GTR	No adjuvant therapy	Died in 12 months after GTR	Yes	+/+	79.6%
3	75	F	GBM IDH-wt, rec, Lynch syndrome	Colon adenocarcinoma	Rt. temporal	5	Multifocal enhancing mass	GTR	CCRT/TMZ	Recur	Yes	+/+	24.1%
4	69	M	GBM IDH-wt, rec, Lynch syndrome	Multiple GI adenocarcinomas, prostatic carcinoma	Rt. occipital	6	Heterogeneous enhancing mass with perilesional edema	GTR	Hypo-CCRT/TMZ	Recur	Yes	+/+	88.8%
5	66	M	GBM IDH-wt, rec	–	Rt. frontal	5.3	Irregular enhancing mass	GTR	Hypo-CCRT/TMZ (Incomplete)	Recur	Yes	+/+	37.6%
6	73	F	GBM IDH-wt, rec	–	Rt. temporal	1.9	Rim enhancing irregular mass	GTR	PO-RT only	Recur	Yes	+/+	85.2%
7	58	M	GBM IDH-wt	–	Posterior fossa^a^	2.2	Enhancing mass	biopsy	CCRT/TMZ	Stationary in 4 months after biopsy	Yes	+/−	83.6%
8	50	F	GBM IDH-wt, Lynch syndrome	–	Lt. thalamus, basal ganglia, mid-brain	4.6	Enhancing mass lesion	GTR	CCRT/TMZ	Stationary in 3 months after GTR	Yes	+/+	63.8%
9	78	M	Gliosarcoma, IDH-wt, rec	MMRD after CCRT	Lt. cerebellum	4	Heterogeneous enhancing mass	GTR	CCRT/TMZ	Recur	Yes	+/+	18.2%
10	11	M	DMG H3 K27M-m, rec	–	Rt. thalamus	3.6	Multiple enhancing solid and cystic mass	GTR	CCRT/TMZ	Recur (×4) and died in 22 months after GTR	No	+/+	41.8%
11	33	M	Astrocytoma, IDH-m, WHO grade 4, rec	MMRD after CCRT	Rt. frontotemporal	8.5	Enhancing tumor	GTR	No adjuvant therapy	Recur and died in 59 months after GTR	No	+/+	43.1%
12	15	M	Astrocytoma, IDH-m, WHO grade 4, rec	–	Rt. frontal	3.4	Enhancing solid and cystic lesion	GTR	CCRT/TMZ	Recur	Yes	+/+	18.8%
13	35	F	Pleomorphic xanthoastrocytoma, Lynch syndrome	–	Rt. frontal, corpus callosum and cingulate gyrus	3.8	Subtle enhancing and cystic change	GTR	No adjuvant therapy	Stationary	Yes	−/−	2.2%

*rec* recurrent, *GBM IDH-wt* glioblastoma IDH-wildtype, *DMG H3 K27M-m* diffuse midline glioma H3 K27M-mutant, *Posterior fossa*^a^ 4th ventricle and right cerebellum and left vermis, *GTR* gross total resection, *PO-RT* postoperative radiotherapy, *MVP* microvascular proliferation, *Op* operation, + present, absent.

### Magnetic resonance imaging (MRI)

**Fig. 2:**
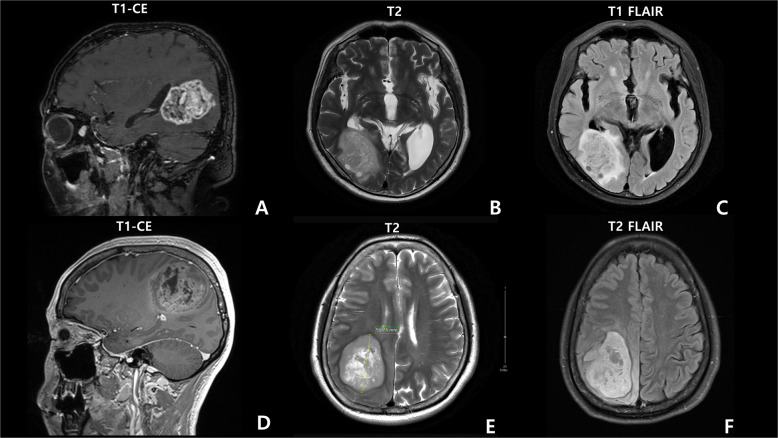
The brain MRI images of case 4 with Lynch syndrome. **A** sagittal T1-weighted (postcontrast), **B** axial T2-weighted, and **C** T2 FLAIR MRI results, showing an ~6 cm-long diameter enhancing mass with perilesional edema in the right occipital lobe. Case 2 (glioblastoma *IDH*-wildtype) **D** sagittal T1-weighted (postcontrast), **E** axial T2-weighted, and **F** T2 FLAIR MRI results, revealing an ~5.7 cm heterogeneous mass in the right parietal lobe and midline shift.

A total of 85% (11/13) of the tumors were located in the supratentorial region (temporal, frontal, frontotemporal, parietal, or occipital lobes or thalamus or corpus callosum and cingulate gyrus or basal ganglia) (Table 1). The remaining two tumors were found in the posterior fossa including the cerebellum. MRI revealed high- and low-signal-intensity masses on T2 and T1 imaging, with rim or heterogeneous enhancement and perilesional edema in the most patients. All MRI findings suggested HGG (Fig. 2).

### Histopathology and immunohistochemistry (IHC) of MMRD brain tumors

Neutral formalin-fixed paraffin-embedded (FFPE) tissues were cut into slices of 3 μm thickness for H&E staining and IHC. Tissue sections were stained with anti-IDH1 R132H (H09) monoclonal antibody (1:100 dilution, Dianova, Hamburg, Germany), anti-ATRX polyclonal antibody HPA001906 (1:300 dilution, ATLAS ANTIBODIES AB, Bromma, Sweden), anti-p53 monoclonal antibody, DO-7 code M7001 (1:1000 dilution, DAKO, Glostrup, Denmark), anti-pHH3 antibody (1:100 dilution, Cell Marque, Rocklin, USA), anti-Ki67 antibody (1:1000 dilution, DAKO, Glostrup, Denmark), anti-H3K27M (K27M) monoclonal antibody (1:1000 Milipore, Temecula, USA), anti-synaptophysin antibody (1:200 dilution, Novocastra, Newcastle, UK), NeuN (1:500 dilution, Millipore, Temecula, USA), anti-BRAF VE1 antibody (1: 200, Spring Bioscience, CA, US), anti-programmed death 1 NAT105 monoclonal antibody (1:50 Cell Marque, Rocklin, USA), anti-programmed cell death 1 ligand 1 22C3 monoclonal antibody (1:50 DAKO, Glostrup, Denmark), and anti-MMR protein antibodies, including anti-MLH1 M1 monoclonal antibody (1: 50, Ventana, Export, USA), anti-MSH2 G219-1129 monoclonal antibody (1: 200, Ventana, Export, USA), anti-MSH6 44 monoclonal antibody (1: 50, Cell Marque, Rocklin, USA), and anti-PMS2 MRQ-28 monoclonal antibody (1:50, Cell Marque). IHC staining was carried out using a standard avidin-biotin-peroxidase method with a BenchMark ULTRA system (Roche Diagnostics). The primary antibodies used in this study are listed in Table 2.

**Table 2:** The primary antibodies used in this study.

Antibody	Dilution	Antigen retrieval	Clone	Source
MLH1	1:50	Ventana CC1 100 °C	M1 (monoclonal)	Ventana, Export, USA
MSH2	1:200	Ventana CC1 100 °C	G219-1129 (monoclonal)	Ventana, Export, USA
MSH6	1:50	Ventana CC1 100 °C	44 (monoclonal)	Cell Marque, Rocklin, USA
PMS2	1:50	Ventana CC1 100 °C	MRQ-28 (monoclonal)	Cell Marque, Rocklin, USA
GFAP	1:200	Ventana CC1 100 °C	6F2 (monoclonal)	DAKO, Glostrup, Denmark
ATRX	1:200	Ventana CC1 100 °C	Polyclonal	Atlas Antibodies AB, Bromma,Sweden
K27M	1:1000	Ventana CC1 100 °C	HH3 (monoclonal)	Milipore, Temecula, USA
Ki67	1:100	Ventana CC1 100 °C	MIB-1 (monoclonal)	DAKO, Glostrup, Denmark
IDH-1	1:100	Ventaan CC1 100 °C	H09 (monoclonal)	Dainova, Hamburg, Germany
P16	1:100	Ventana CC1 100 °C	E6H4 (monoclonal)	Ventana, Export, USA
P53	1:1000	Ventana CC1 100 °C	DO7 (monoclonal)	DAKO, Glostrup, Denmark
pHH3	1:100	Ventana CC1 100 °C	Polyclonal	Cell Marque, Rocklin, USA
Synaptophysin	1:200	Bond H2O ER2 200 °C	27G12 (monoclonal)	NOVO, Newcastle, UK
NeuN	1:500	Ventana CC1 100 °C	A60 (monoclonal)	Millipore, Temecula, USA
BRAF	1:200	Ventana CC1 100 °C	VE1 (monoclonal)	Spring Bioscience, CA, US
PD1	1:50	Ventana CC1 100 °C	NAT105 (monoclonal)	Cell Marque, Rocklin, USA
PD-L1(22C3)	1:50	Ventana CC1 100 °C	22C3 (monoclonal)	DAKO, Glostrup, Denmark

*MLH1* MutL Protein Homolog 1, *MSH2* Mut-S-homolog-2, *MSH6* Mut-S-homolog-6, *PMS2* postmeiotic segregation increased 2, *GFAP* glial fibrillary acidic protein, *ATRX* alpha thalassemia associated mental retardation X, *K27M* Histon lysin27methionine, *IDH-1* isocitrate dehydrogenase 1, *pHH3* phosphorylated Histone H3, *NeuN* neuronal nuclear protein,  *PD-1* programmed death 1, *PD-L1* programmed cell death 1 ligand 1.

**Fig. 3:**
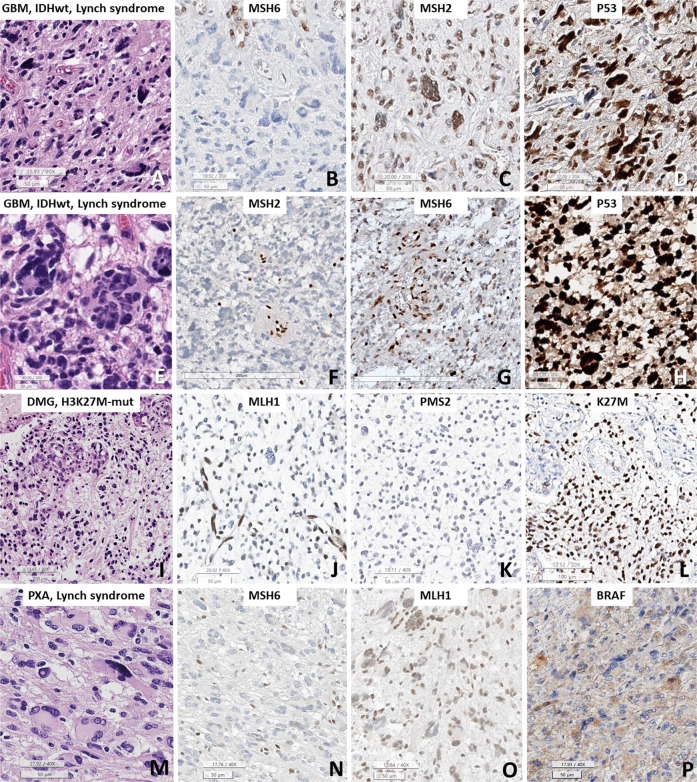
The immunohistochemical results of MMRD brain tumors. **A**–**D** GBM *IDH*-wildtype with Lynch syndrome and *MSH6* mutation (Case 3), **E**–**H** GBM *IDH*-wildtype with Lynch syndrome and *MSH2* mutation (Case 4), **I**–**L** diffuse midline glioma *H3* K27M-mutant (DMG) (Case 10), and (M-P) PXA with *MSH6* mutation (Case 13). **A**, **E** Bizarre multinucleated giant cells (Cases 3 and 4) were predominant. **B**, **C** *MSH6*-mutant tumors showed loss of MSH6 expression but no loss of MSH2, as expected. **F**, **G** The *MSH2*-mutant case (Case 4) showed loss of MSH2 protein but heterogeneous loss of MSH6, suggesting that the partner protein was not completely lost. **D**, **H** P53 staining showed overexpression in both Case 3 and Case 4. **I** The DMG *H3* K27M-mutant showed no bizarre multinucleated giant cells but did show microvascular proliferation. **J**, **K** Both MLH1 and PMS2 loss were present. **L** K27M staining showed nuclear positivity. **M** The case with PXA with Lynch syndrome showed marked multinucleated giant cells and vacuolar cells and stroma. **N** There was loss of MSH6 expression, but the expression of its partner protein (MLH1) was retained. **P** BRAF VE1 staining was positive in the tumor cells. (**A**, **E**, **I**, **M**: H&E; **C**, **F**: MSH2; **B**, **G**, **N**: MSH6;: p53; **J**, **O**: MLH1; **K**: PMS2; **L**: K27M; and **P**: BRAF. Bar size: **A**–**D**, **H**, **J, K, M**–**P**: 50 micrometers; E: 20 micrometers; **F**, **G**: 200 micrometers; **I**, **L**: 100 micrometers).

We used a proper positive control. Most cases had internal positive controls on the slides (Fig. 3), and for the negative control, we omitted the primary antibodies. The Ki67 labeling index was calculated on virtual Leica Biosystems slides (Aperio ScanScope system) using the SpectrumPlus Nuclear Algorithm n9 image analyzer. The positive controls for PD1 and PD-L1 were a known PD1/PD-L1-positive tumor and positive lymphocytes.

Complete loss of expression of MLH1, PMS2, MSH2, or MSH6 in tumor cell nuclei on IHC indicated loss of the respective protein, and heterogeneous loss of expression was defined as a mixture of areas with loss of expression and retained expression. According to Graham et al.’s paper, heterogeneous MSH6 loss is uncommon but exists and is usually caused by MSI and instability of the MSH6 exon 5 polycytosine tract but is not associated with a germline MSH6 mutation^19^.

In the in vivo state, MLH1/PMS2 and MSH2/MSH6 form two functional pairs. When either MLH1 or MSH2 is lost, the partner protein is destabilized and degraded, resulting in the loss of the partner MMR protein. However, the opposite is not true; the absence of PMS2 or MSH6 does not affect stability because MLH1 and MSH2 can bind to and stabilize other molecules^20^. Therefore, we carefully examined the protein expression of these pairs.

Histopathology was reviewed by two pathologists (HK and SHP) according to the histopathological criteria defined by the 2021 WHO classification^21^ and cIMPACT-NOW updates^22^.

### DNA and RNA extraction for next-generation sequencing (NGS), *MGMT* promoter (*MGMTp*) methylation studies, and microsatellite instability studies

Representative areas of the tumor from FFPE tissue on H&E-stained sections with at least 90% tumor cell content were outlined for macrodissection. DNA/RNA extraction was performed from these FFPE tissues using the Maxwell^®^ RSC DNA/RNA FFPE Kit (Promega, USA) according to the manufacturer’s instructions. For MSI polymerase chain reaction (PCR) studies using Bethesda’s five-marker panel and for the germline MMR mutation study, paired tumor and normal tissue samples were used for genomic DNA extraction. Definitively normal tissue adjacent to the brain tumor was used as the normal counterpart. If it was difficult to find normal tissue on the H&E-stained slide, we verified that it was normal tissue with Ki-67, EGFR, or TP53 immunostaining. If no normal tissue was present in the brain tumor biopsy sample, biopsied extracranial normal tissue or blood was used as the normal counterpart. Genomic DNA was subjected to PCR with fluorescently labeled oligonucleotide primers for five microsatellite loci (BAT25, BAT26, D2S123, D5S346, and D17S250), followed by capillary electrophoresis on an ABI 3100 genetic analyzer (Applied Biosystems, Foster, CA, US). The instability of the investigated loci was defined as a change in the length of the PCR product in a tumor sample compared to the length of the PCR product in the paired normal sample. MSI status was classified as MSI-H if the sample showed instability at two or more microsatellite loci, MSI-low (MSI-L) if the sample showed instability at one locus, and microsatellite stable (MSS) if there was no instability.

### NGS and pipelines of analysis of the somatic mutations

NGS studies were performed with tumor DNA extracted from FFPE tumor tissue and NEXTSeq Dx505 using a customized brain tumor gene panel (The FIRST brain tumor panel established by the Department of Pathology, SNUH, and approved by the Korea Food and Drug Administration), which assesses 207 brain tumor-associated genes and 54 fusion genes, including 4 MMR genes (Supplementary Table 2). Fusion genes were sequenced using RNA. Somatic mutations were detected using the Genome Analysis Toolkit (GATK) Mutect2 v4.1.4.1. with default parameters^23^. To avoid germline variant contamination, we used the gnomad.hg19.vcf Genome Aggregation Database (gnomAD)^24^ and 1000 g_pon.hg19.vcf files, which include a normal panel for 1000 genomes. The files were provided by the GATK resource bundle. After calling somatic mutations, all variants were annotated by ANNOVAR (https://doc-openbio.readthedocs.io/projects/annovar/en/latest/)^25^.

We extracted recent 20 cases of IDH-mutant and 60 cases of IDH-wildtype grade 4 gliomas from our hospital NGS data and we compare the number of mutations between MMRD gliomas and non-MMRD gliomas.

### Sanger sequencing for germline study

DNA was extracted from FFPE and blood for germline study of MMR genes using a DNA extraction kit (Promega, A2352). Gene-specific primers were added to 20 µl reaction PCR premix (Bioneer, K-2012). Primers were designed using Primer3 (https://bioinfo.ut.ee/primer3-0.4.0/) (Supplementary Table 3)^26^. PCR products were analyzed to validate gene mutations using Sanger sequencing.

### R programming

Clinical information, mutations, and copy number variations were summarized with Oncoprint data, which were generated using the R package ComplexHeatmap (version 2.7.6.1002, R version 4.0.3)^27^. Progression-free survival (PFS) and overall survival (OS) plots were generated using the R packages Survival (version 3.2-11, R version 4.0.3) and Survminer (version 0.4.9, R version 4.0.3).

### Survival analysis

Kaplan–Meier survival analysis was performed, and *IDH*-wildtype GBMs and *IDH*-mutant WHO grade 4 astrocytoma with intact MMR were compared. The control cases were previously established cohort in Park et al.’s paper^28^. PFS was defined as the time from first surgery for the brain tumor to disease progression, while OS was defined as the time from the first surgery for the brain tumor to death.

## Results

### Imaging, histopathology, and immunohistochemistry

The locations of the MMRD brain tumors were the temporal (3), frontal (3), frontotemporal (1), parietal (1), and occipital (1) lobes, thalamus (2), cerebellum (1), and posterior fossa (1) (Table 1). On MRI of HGGs, the tumors showed high and low signal intensity on T2 and T1 imaging, respectively, with rim or heterogeneously enhanced parts (Fig. 2) of variable sizes, ranging from 1.9 to 8.5 cm.

Histopathologically, 10 tumors (77%), which were all HGGs, showed marked bizarre multinucleated giant cells (Table 1, Fig. 3A, E). The remaining three tumors did have somewhat pleomorphic nuclei but did not have numerous multinucleated giant cells (Table 1, Fig. 3I). Microvascular proliferation was observed in 12 cases (92%), and necrosis was observed in 10 cases (77%).

Among the four *MLH1*-mutant tumors, complete loss of both MLH1 and PMS2 IHC in the tumor cells was present in three cases (Patient #1, 9, and 10), but one case showed complete loss of MLH1 and heterogeneous loss of the partner protein PMS2 (Patient #11). Four *MSH2*-mutant tumors showed complete loss of MSH2 IHC but a heterogeneous loss of the partner protein MSH6 (Patient #2, 4–6) (Fig. 3F, G) (Table 3). Three (Cases #3, 12, and 13) of four *MSH6*-mutant tumors had MSH6 loss only (Fig. 3C, G), but both MSH6 and PMS2 losses were also found in one *MSH6*-mutant tumor (Patient #8). These results were expected because it is already known that MSH2 loss can result in a heterogeneous loss of the partner protein MSH6 as a result of MSI and instability of the MSH6 polycytosine tract, but *MSH6*-mutant tumors are known to have no partner protein loss^19^.

**Table 3:** The immunohistochemical and molecular studies including MMR genes, MMR protein, and MSI status in our cases.

#	Diagnosis	Mutant MMR gene, variant allele frequency	MLH1/PMS2	MSH2/MSH6	MSI	7p + &10q-/EGFR amplification/PTEN loss	BRAF mutation	TERT promoter mutation	ATRX/IDH1/K27M IHC	PD1/PDL1	MGMT
1	GBM IDH-wildtype	MLH1, p.Ser685Phe, 36.1%, likely pathogenic	Loss/Loss	No loss/No loss	MSS	−/−/p	−	Present	+/−/−	−/−	M
2	GBM IDH-wildtype	MSH2, p.Leu372*, 94.7%, pathogenic	No loss/No loss	Loss/Loss^a^	MSI-H	−/−/p	−	−	+/−/−	−/−	UM
3	GBM IDH-wildtype, Lynch syndrome	MSH6, p.Ser602*, 48.6%, pathogenic	No loss/No loss	No loss/Loss	MSI-H	−/−/−	−	−	+/−/−	−/−	UM
4	GBM IDH-wildtype, Lynch syndrome	MSH2, p.Tyr405*, 92.9%, pathogenic	No loss/Loss	Loss/Loss^a^	MSI-H	−/−/−	−	−	+/−/−	−/−	M
5	GBM IDH-wildtype	MSH2, p.Gln510*, 16.3%, pathogenic	No loss/No loss	Loss/Loss^a^	MSI-L	−/−/p	−	Present	+/−/−	−/−	M
6	GBM IDH-wildtype	MSH2, splicing, 14.5%, pathogenic	No loss/No loss	Loss/Loss	MSS	−/−/−	−	Present	+/−/−	A few (+) /weak (+)	M
7	GBM IDH-wildtype	PMS2, p.Thr337fs, 57.02%, pathogenic	No loss/Loss	No loss/No loss	MSS	−/−/−	−	−	+/−/−	−/−	UM
8	GBM IDH-wildtype, Lynch syndrome	MSH6, p.Phe1088fs, 23.86%/p.Gln889fs, 44.46%, pathogenic	No loss/Loss	No loss/Loss	MSI-H	−/−/−	−	−	+/−/−	A few (+) /weak (+)	UM
9	Gliosaroma IDH-wildtype	MLH1, p.Arg127Ile, 5.0%, pathogenic	Loss/Loss	Loss^a^/No loss	MSS	−/−/−	−	−	+/−/−	−/weak (+)	M
10	DMG H3 K27M-altered	MLH1, p.Ala353fs, 54.3%, likely pathogenic	Loss/Loss	No loss/No loss	MSI-H	−/−/p	−	−	Loss/−/+	−/−	UM
11	Astrocytoma, IDH-mutant, WHO grade 4	MLH1, p.Arg687Trp, 87.1%, pathogenic	Loss/Loss^a^	No loss/No loss	MSI-H	−/−/−	−	−	Loss/+/−	−/−	M
12	Astrocytoma, IDH-mutant, WHO grade 4	MSH6, p.Arg1172fs, 80.5%, pathogenic	No loss/No loss	No loss/Loss	ND	−/−/−	−	−	+/+/−	−/−	UM
13	Pleomorphic xanthoastrocytoma, Lynch syndrome	MSH6, p.Arg1334Gln, 55.06%, pathogenic	No loss/No loss	No loss/Loss	MSS	−/−/−	+ (V600E)	−	+/−/−	−/−	M

*+ or p* positive, *−* negative, *Loss*^*a*^ Heterogeneous loss of expression, *M* MGMT promoter-methylated, *UM* MGMT promoter-unmethylated, *MSS* microsatellite stable, *MSI-H* microsatellite instability-high, *MSI-L* microsatellite instability-low, *weak (+)* weak positive (+/3) in 1% of tumor cells, *A few (+)* positive in up to 4/HPF, *7p+&10q-* the concurrent gain of whole chromosome 7 and loss of whole chromosome 10, *ND* not done.

PDL-1 was weakly positive in 1% of tumor cells in three patients (cases #6, #8, and #9), but it was not expressed in the other cases. PD-1 was positive in a few immune cells in case #6 and #8 (positive in up to 4 cells/HPF) and was not expressed in the other tumors (Table 3). The Ki-67 labeling index was relatively high (Table 1).

### Molecular analysis and next-generation sequencing (NGS) study

**Fig. 4:**
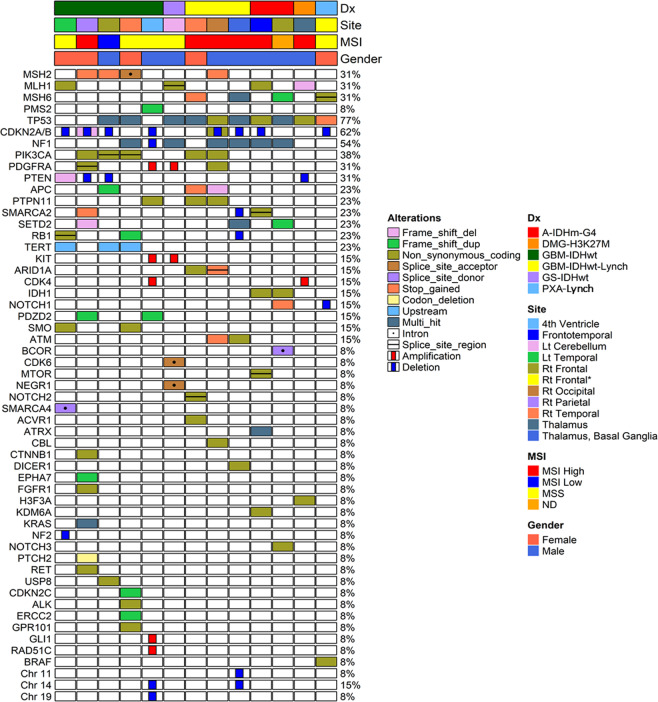
The OncoMap of clinicopathological data for 13 MMRD brain tumor cases. Clinicopathological and molecular genetic features and NGS results of 13 cases listed in the OncoMap System (*GS* gliosarcoma, *Dx* diagnosis).

MSI-H was found in 50% (6/12) of patients (Supplementary Fig. 1) including all four Lynch syndrome-associated cases, MSI-L was found in one patient (8%), and five patients exhibited MSS. The MSI study could not be performed in the remaining one case because there was no normal tissue (Table 3). *MGMT*p methylation was found in 53.8% (7/13) of tumors, and among GBMs and gliosarcomas, 55.5% (5/9) of them had *MGMTp* methylation. The NGS studies found MMR gene mutations as well as multiple pathogenic mutations and variants of uncertain significance (Fig. 4). The variants of *MLH1* were p.Ser685Phe/c.2054 C>T, p.Ala353fs/c.1057delG, p.Arg127Ile/c.380 G>T, and p.Arg687Trp/c.2059 C>T. The variants of *MSH2* were p.Leu372*/c.1115 T>A, p.Tyr405*/c.1215 C>A, p.Gln510*/c.1528 C>T, and splicing/c.1511-1 G>A. The variants of *MSH6 were* p.Ser602*/c.1805C>G, p.Arg1172fs/c.3514dupA, p.Arg1334Gln/c.4001 G>A, p.Gln889fs/c.2665dupC, p.Phe1088fs/c.3261dupC, and p.Phe1088fs/c.3261dupC. one GBM showed a *PMS2*, variant (p.Thr337fs/c.1009dupA).

The MMR gene mutations were verified by IHC (Fig. 3). Notably, the *MSH2* p.Tyr405* mutation found in patients with Lynch syndrome is a known germline variant but has never been reported as a somatic mutation in the OnkoKB and Cosmic databases^29^. *TP53* showed the highest frequency of pathogenic variants, with variants found in 10 tumors [p.Arg273His/c.818 G>A (in 2 gliomas), p.Arg273Cys/c.817 C>T, p.Arg175His/c.524 G>A (in 2 gliomas), p.Arg248Gly/c.742 C>G, p.Arg342*/c.1024 C>T, p.Arg248Trp/c.742 C>T, p.Val173Leu/c.517 G>T, p.Arg213Gln/c.638 G>A, p.Gly245Ser/c.733 G>A, p.Arg213*/c.637 C>T, p.Arg267Trp, c.799 C>T, p.Arg248Gln, c.743 G>A]. Other frequent pathogenic variants were *CDKN2A/2B* hemizygous deletion and mutations (p.Ala36fs c.106delG and p.His83Tyr c.247 C>T) found in eight tumors, and *NF1* mutations (single or both alleles; p.Ser82Phe/c.245 C>T, p.Trp426*/c.1278 G>A, p.Trp1559*/c.4677 G>A, splicing c.6642 + 1 G>A, p.Asn78fs/c.233dupA, p.Arg2258*/c.6772 C>T, splicing c.1185 + 1 G>T, p.Pro1421Gln/c.4262 C>A, p.Ile679fs/c.2033dupC, p.Arg1611Gln/c.4832 G>A, p.Arg1611Trp/c.4831 C>T, p.Trp2369*/c.7107 G>A, and p.Arg1769*, c.5305 C>T, p.Cys1960fs, c.5878delT, p.Cys1960fs c.5878delT) and *NF1* deletion were found in 7 tumors (Supplementary Table 1).

**Fig. 5:**
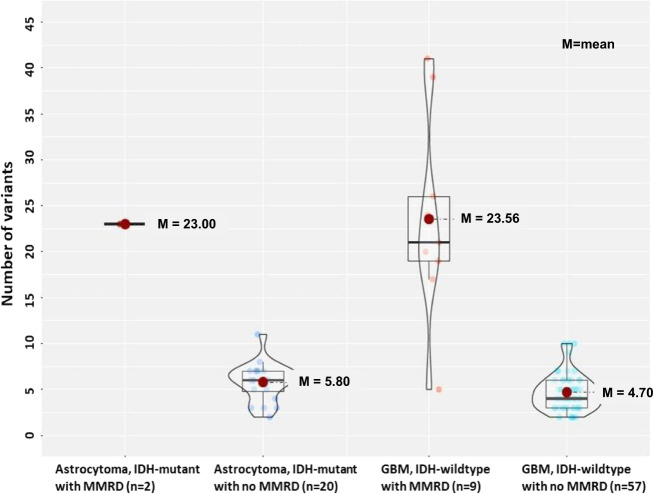
The box plot of the number of nonsense mutation of high-grade gliomas with/without MMRD. MMRD high-grade gliomas had higher number of nonsense mutation than non-MMRD gliomas; The average number of mutations in astrocytoma IDH-mutant with MMRD and without MMRD is 23.0 and 5.8, respectively. The average number of mutations in glioblastoma, IDH-wildtype with MMRD and without MMRD was 23.6 and 4.7, respectively.

The number of nonsense mutation was higher in MMRD-HGG (average 23.0–23.6) and than HGGs without MMRD (average 4.7–5.8) (Fig. 5). Of the 3 MMRD gliomas with low mutation numbers (5 SNPs), GBM IDH-wt (Case #7) had many copy number aberrations, but the remaining DMG (Case #7) and PXA (Case #13) did not have many copy number aberrations, eventhough they had MMRD (Supplementary Table 3).

Germline studies of MMR genes by Sanger sequencing revealed germline mutations in three cases (Cases #3, #8, and #13, Supplementary Fig. 2). In one remaining patient, the germline study could not be performed because there was no normal tissue or blood. This patient had a solitary brain tumor only, and additional clinicopathological reviews suggested sporadic MMRD brain tumors.

### Treatment, follow-up of patients, and survival analysis (PFS and OS)

After the surgery, nine patients with GBM and DMG were treated with CCRT with temozolomide (TMZ), and one patient with GBM (Case #6) was treated with postoperative radiotherapy (PO-RT) only. However, the remaining three patients did not receive adjuvant therapy (Table 1). The intestinal carcinoma of a Lynch syndrome patient (Case 4) who had been treated with postoperative adjuvant 5-fluorouracil, leucovorin, and oxaliplatin did not recur for 8 years. Instead, this patient’s MMR-intact prostatic adenocarcinoma metastasized to multiple bones, including the rib, thoracic spine, sacrum, and pelvic bones, during the last 7 years, despite radiation therapy, chemotherapy (docetaxel and abiraterone/prednisone), and androgen deprivation therapy.

Four patients (31%) died from diseases, 10 patients (77%) had recurrences of tumors and the remaining 3 patients did not have enough follow-up period (~2 months in all 3 patients). Patient #1 recurred after 1 year of treatment, despite gross total resection (GTR) of the tumor plus CCRT and gamma knife stereotactic radiosurgery, and died in 57 months after the initial surgery. Patient #2 died at 12 months after GTR with no adjuvant therapy, and patient #10 died at 22 after GTR and CCRT with recurrences. Case #11 with astrocytoma *IDH*-mutant recurred in 39 months and died in 59 months after GTR. One patient (Case #12) with sporadic astrocytoma *IDH*-mutant WHO grade 4 did not recur. PFS of the patients with recurrent tumors was 1 month to 42 months.

**Fig. 6:**
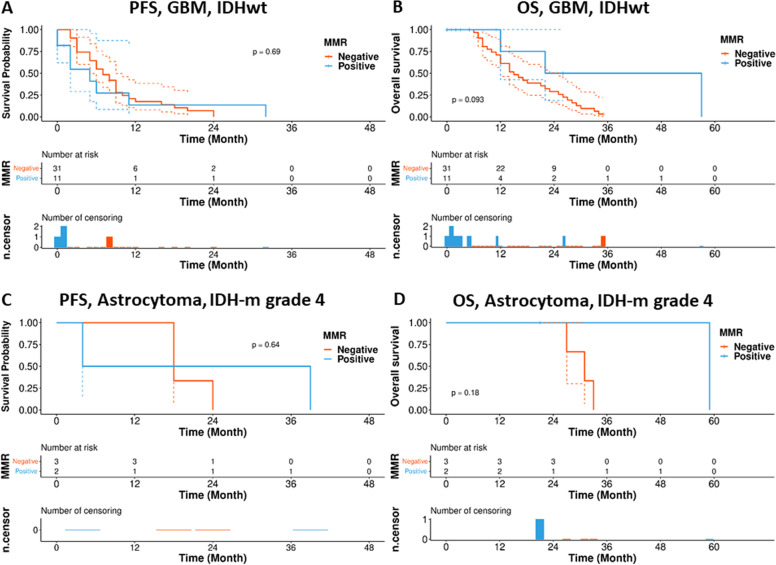
The Kaplan–Meier plot of progression-free survival (PFS) and overall survival (OS) for high-grade gliomas with/without MMRD. Kaplan–Meier analysis of progression-free survival (PFS) and overall survival (OS) for (**A, B**) *IDH*-wildtype glioma with/without MMRD and (**C, D**) *IDH*-mutant glioma with/without MMRD. **A** *P* = 0.69; **B** *P* = 0.093; **C** *P* = 0.64; **D** *P* = 0.18.

Kaplan–Meier survival analysis showed a trend for lower PFS in patients with MMRD-HGG than those in patients without MMRD-HGG (*p* = 0.69, *p* = 0.64) (Fig. 6A, C). However, all the results did not have a statistical significance due to the small number of cases, short follow-up duration, and also better patient care. (*P*>0.05) (Fig. 6B, D).

## Discussion

MMRD brain tumors are very rare, accounting for ~2% of primary brain tumors, and are also histopathologically diverse, including GBM, astrocytoma, oligodendroglioma, gliosarcoma, anaplastic PXA, medulloblastoma, and neuroblastoma^30^. Among them, GBM and high-grade astrocytoma are the most common sporadic or inherited MMRD brain tumors^11,31^. The proportion of MMRD primary brain tumors in the 740 brain tumors studied with NGS in our hospital for 3 years was ~2%, which include 4 Lynch syndrome-related and 2 CCRT-induced MMRD brain tumors.

In line with our results, the inactivation of MMR genes has been identified in both *IDH*-mutant and *IDH*-wildtype gliomas^32^. Pediatric HGGs, such as DMG H3 K27M-altered, medulloblstoma, and anaplastic PXA, have also been reported to have MMD^33^. Lynch syndrome is the most common form of hereditary colorectal cancer, accounting for 2–7% of all cases of colorectal cancer^34^. Extracolonic tumors of Lynch syndrome include cancers of the small bowel, pancreas, urinary tract, prostate, and brain^11,34^. The presence of monoallelic germline MMR gene defects is essential for the diagnosis of Lynch syndrome. Constitutional MMRD syndrome has biallelic germline mutations in *MMR* genes, is autosomal recessive, and usually has severe nuclear pleomorphism and multinucleated giant cells, as is seen in Lynch syndrome-associated gliomas^31,35^. Our Lynch syndrome patients had a germline *MSH2* mutation (p.Tyr405*/c.1215 C>A) and *MSH6* mutation (p.Ser602*/c1805C>G, p.Arg1334Gln/c.4001 G>A, p.Phe1088fs/c.3261dupC, p.Gln889fs/c.2665dupC), which have previously been reported in a Lynch syndrome patients^29,36^. However, *POLE* and *MUTYH* gene mutations can also be diagnostic for Lynch syndrome^34^.

We obtained MMRD-associated genes from cBioportal, namely, *CHEK1, CHEK2, RAD51, BRACA1, BRACA2, MLH1, MSH2, ATM, ATR, MDC1, PARP1*, and *FANCF*. Among them, *MSH2* defects were found in 0.2% of the GBMs and 0.6% of all primary brain tumors in TCGA data. To explore MMRD-associated primary brain tumors, we downloaded the gene profiles of primary brain tumors from cBioportal (TCGA database) (file:///D:/L/Lynch%20synd%20MMRD%20br%20T/oncoprint.svg). The *MSH2* mutation-associated MMRD brain tumors included 43 gliomas, including GBM (*n* = 20), oligodendroglioma (*n* = 9), anaplastic oligodendroglioma (*n* = 6), diffuse astrocytoma (*n* = 5), anaplastic astrocytoma (*n* = 2), and oligoastrocytoma (*n* = 1). Missense mutation was the most common type of mutation in MMR genes, found in 65.1% (28/43) of cases, and nonsense and splice mutations were found in 21% and 12% of cases, respectively. There was one case each with frameshift and insertion mutations.

GBMs are usually chromosomally unstable, thus commonly have chromosomal aberrations and aneuploid DNA content^37^. Unlike the conventional GBM *IDH*-wildtype^22,38^, our nine cases of MMRD GBM *IDH*-wildtype did not have the concurrent gain of chromosome 7 and loss of chromosome 10 or *EGFR* amplification. Instead, MMRD gliomas had mutations in *TP53, NF1, and PIK3CA*, amplification of *PDGFRA*, and deletion of *CDKN2A/2B*. Variable *PTEN* alteration, including frameshift mutation (*n* = 1) and hemizygous deletion (*n* = 2) was found in 33% (3/9). *TERT promoter (TERTp)* mutation (C250T and C228T) was present in 33% (3/9) (Table 3, Fig. 4). In our study, there were five cases with MSS despite MMRD but all our Lynch syndrome-associated HGG had MSI-H.

Gliosarcomas *IDH*-wildtype usually have *TP53* and *PTEN* mutations and *CDKN2A* deletions, but *EGFR* amplification is rare^39^. In this study, the gliosarcoma *IDH*-wildtype had two *TP53* mutations (p.Arg248Trp, c.742 C>T and p.Val173Leu, c.517 G>T) and *NF1* mutation (splicing, c.1185+1 G>T and p.Pro1421Gln, c.4262 C>A), and *PDGFRA* amplification; however, neither *PTEN* mutation nor *CDKN2A* deletion was present. DMG *H3* K27M-mutant can have *TP53* mutation (~50% of cases) and *ATRX* mutation (loss of expression; 10–15% of cases)^40^. Our MMRD DMG *H3* K27M-mutant also had *TP53* mutation (p.Arg273His, c.818 G>A, VAF 43%) and *ATRX* mutation. Astrocytoma *IDH-*mutant WHO grade 4 can have *CDKN2A*/B homozygous deletion^41^, and this gene deletion was found in one out of two astrocytoma *IDH-*mutant in this study. Our PXA case had *BRAF* mutation (p.Val600Glu, c.1799T>A).

Inactivating mutations of *TP53* and chromosomal instability following the loss of MMR function are common genetic abnormalities^37^. However, because colorectal carcinomas usually have diploid or near-diploid DNA content with a few chromosomal aberrations, colorectal carcinomas with MMRD usually do not have inactivating mutations of *TP53* and chromosomal instability^37^. The MMRD *IDH*-wildtype and *IDH-*mutant HGGs in this study commonly had additional pathogenic missense mutations of the *TP53, NF1, PIK3CA*, deletion or mutation of *CDKN2A/2B*, amplification or mutation of *PDGFRA*. In addition, copy number aberrations of various genes and many other VUS, suggesting a high TMB.

Because of the presence of mutations in the *TP53, NF1*, and *ATM* genes, concomitant or underlying Li-Fraumeni, neurofibromatosis type 1, or ataxia-telangiectasia cancer syndrome needed to be ruled out. However, these cancer-predisposing syndromes require germline mutations for diagnosis, and the associated tumor types are different from those seen in our Lynch syndrome cases; Li-Fraumeni syndrome-associated cancers are usually sarcomas, breast cancers, brain tumors, and leukemias^42^. Brain tumors of Li-Fraumeni syndrome can appear as low-grade gliomas or HGGs^43^. Patients with neurofibromatosis type 1 typically have neurofibroma, optic nerve glioma, or malignant peripheral nerve sheath tumor, and patients with ataxia-telangiectasia syndrome usually have non-Hodgkin lymphoma and leukemia^42^.

Hypermutation can occur in recurrent tumors after TMZ treatment via MMRD and MSI-H-related mechanisms, whereas these alterations are extremely rare in primary brain tumors^44^. MMRD is often associated with MSI, and it is one of the mechanisms behind acquired resistance to the alkylating chemotherapeutic agent TMZ in gliomas^44^.

MMRD is also related to TMB and neoantigen loads, therefore, can be a target of immunotherapy. Generally, MMRD tumors have MSI-H^17^. MSI-H is generally uncommon in sporadic brain tumors, but if it is present, it may represent MMR gene germline mutation carriers^45^. Although one study reported that MSI is rare in Lynch syndrome-associated brain tumors^46^, it can occur as a result of the loss of MMR function^17^. Our 4 Lynch associated HGG had MSI-H. The TMB could not be verified in our cases due to the limitations of the targeted gene panel (207 brain tumor-targeted genes and 54 fusion genes), but most of our cases possibly had TMB because of many pathogenic and likely pathogenic mutations and VUS. However, three cases (1 GBM IDHwt, 1 PXA, 1 DMG) had less than five SNPs with variable copy number aberrations (Fig. 5, Supplementary Table 1).

Among MMRD-associated tumors, the number of methylated genes is known to be the lowest in brain tumors and the highest in colorectal cancers^47^. The methylation of *MGMTp* is known to occur in approximately half of MMR-intact gliomas; therefore, the incidence of *MGMTp* methylation in MMRD gliomas could be similar to that in MMR-intact gliomas^48^. Methylation of *MGMTp* was observed in about half (53.8%) of our cases.

According to a recent study, replication repair-deficient (RRD) HGGs have a global methylation pattern distinct from that of replication repair-intact HGGs^49^. This methylation pattern varies according to key driver mutations; for example, the *IDH1* R132H and *H3F3A* K27M mutations, and the location of the tumor. Even in the same RRD HGG case, the methylation patterns of the initial tumor and recurrent tumor can be different. In addition to the methylation pattern, hypomethylation in specific gene pathways associated with critical cellular functions occurs in RRD HGGs, which can be used as a target for treatment. Therefore, methylation patterns should be studied to help classify and treat MMRD gliomas.

Loss of nuclear MMR protein was 100% correlated with MMR gene mutation in our cases, suggesting that IHC is sufficient to identify MMRD in brain tumors. MLH1/PMS2 and MSH2/MSH6 form two functional pairs in vivo. Loss of MLH1 or MSH2 destabilizes and degrades the partner protein, so MLH1/PMS2 and MSH2/MSH6 pair losses are common^50^. However, the opposite is not true; the absence of PMS2 or MSH6 does not affect the stability of the partner protein, because MLH1 and MSH2 can bind to and stabilize other molecules^20^. Therefore, the expression of these protein pairs must be carefully investigated. Most of our cases showed these patterns.

MMRD is often associated with MSI-H and TMB, which can contribute to poor outcomes^1,2^. Unexpectedly, PFS and OS were not significantly different between glioma patients with and without MMRD (*P*>0.05) (Fig. 6), possibly due to the limited number of MMRD cases and the short follow-up duration. To determine the biological behavior of MMRD brain tumors, more large-scale, well-designed prospective studies are required. Randomized controlled trials are necessary to improve the treatment of MMRD patients.

PD1/PDL1 IHC staining was almost negative in our cases. Identifying patients with MMRD brain tumors is important for appropriate treatment strategies for patients with sporadic MMRD tumors and family members at risk for Lynch syndrome or CMMRD. Since the PD1/PDL-1 IHC staining was negative in most of our MMRD brain tumors, other options, such as direct identification of MMRD via NGS or IHC studies in primary brain tumors, may be needed to determine the indications for immunotherapy. These MMRD gliomas could be sensitive to immunotherapy but resistant to TMZ^51^.

In conclusion, we analyzed nine sporadic MMRD and four Lynch syndrome-associated gliomas in this study, representing a rare event, about 2.0% of the primary brain tumor cases in our hospital. Most (92%) cases were grade 4 except for one PXA, which was WHO grade 2. MMRD developed after CCRT in 2 cases, which were both *IDH*-mutant and *IDH*-wildtype gliomas. These MMRD gliomas contained many pathogenic and benign or likely benign mutations and VUS, suggesting high TMB, but one GBM, DMG, and PXA did not have TMB despite the MMRD. Half of our MMRD-gliomas and all Lynch syndrome-associated GBMs had MSI-H. Genetic profile of MMRD-associated GBMs was different from that of conventional GBMs. The MMRD GBM did not have EGFR amplification, PTEN homozygous deletion, or concurrent 7p gain and 10q loss. *TERT*p mutation was found in only 33% (3/9) of *IDH*-wildtype MMRD GBMs. *MGMTp* methylation was found in 54% of our MMRD cases. The PFS of our MMRD patients had a tendency of early recurrence. More studies are needed in the form of clinical trials of immunotherapy for MMRD brain tumors.

## Supplementary information

Supplementary Information

## Data Availability

The datasets used and/or analyzed during the current study are available from the corresponding author on reasonable request.
